# Use of an Aqueous Solution of Tartar

**Published:** 1851-11

**Authors:** C. Cloc


*On the External Use of an Aqueous Solution of Tartar Emetic.* By Dr C. Cloc.—The author, in a paper published in the Gazzetta Medica Toscana, reports the effects of the above application in various painful and inflammatory affections, both acute and chronic, as, for example, in acute arthritis, in an inflammatory swelling of the left elbow, in erysipelas of the face supervening during convalescence from small pox, in metastatic cynanche parotidea, in a leucorrhœa of long standing, which was cured by injections into the vagina of a solution of tartar emetic, &c. It must be observed that the topical use of the remedy was combined with the ordinary antiphlogistic treatment, and with general and local blood-letting. Nevertheless, its effects were rapid and evident. The author draws from his experience the following conclusions:

1. Tartar emetic, dissolved in a large quantity of water, and applied externally as a fomentation, is capable of subduing superficial inflammation, and is preferable to all other local antiphlogistics.

2. The solution employed by him consisted of ten grains only of tartar emetic in a pound of water, although a greater proportion might be employed.

3. The cloths should be well moistened and frequently changed.

4. As this cannot be done during the night, a small pledget wet with plain water is then to be substituted, so as to dissolve any particles of the salt which may happen to be left on the surface by evaporation.

5. The cloths should be of linen, and they should be folded double.

6. The effects are more rapidly produced if the cuticle be previously removed.

7. If the solution be applied to a blistered surface, a dry, smooth, shining crust is formed, without producing pain to the patient.

8. No inconvenience was produced by the application, even when continued for fifteen days or more, nor did it give rise to any gastro-enteric or general disturbance, whether employed upon the sound skin or over leech-bites, or where the cuticle had been removed.—*L'Osservatore Medico di Napoli*, April 15, 1851.

